# Lignocellulosic Biomass as Source for Lignin-Based Environmentally Benign Antioxidants

**DOI:** 10.3390/molecules23102664

**Published:** 2018-10-16

**Authors:** Abla Alzagameem, Basma El Khaldi-Hansen, Dominik Büchner, Michael Larkins, Birgit Kamm, Steffen Witzleben, Margit Schulze

**Affiliations:** 1 Department of Natural Sciences, Bonn-Rhein-Sieg University of Applied Sciences, von-Liebig-Str. 20, D-53359 Rheinbach, Germany; abla.alzagameem@h-brs.de (A.A.); basma.elkhaldi-hansen@h-brs.de (B.E.K.-H.); dominik.buechner@h-brs.de (D.B.); mclarki2@ncsu.edu (M.L.); steffen.witzleben@h-brs.de (S.W.); 2 Brandenburg University of Technology BTU Cottbus-Senftenberg, Faculty of Environment and Natural Sciences, Platz der Deutschen Einheit 1, D-03046 Cottbus, Germany; b.kamm@kplus-wood.at; 3 Department of Forest Biomaterials, North Carolina State University, 2820 Faucette Drive Biltmore Hall, Raleigh, NC 27695, USA; 4 Kompetenzzentrum Holz GmbH, Altenberger Strasse 69, A- 4040 Linz, Austria

**Keywords:** antioxidant activity, biomass, Folin-Ciocalteu assay, kraft lignin, lignocellulose feedstock, organosolv, total phenol content

## Abstract

Antioxidant activity is an essential aspect of oxygen-sensitive merchandise and goods, such as food and corresponding packaging, cosmetics, and biomedicine. Technical lignin has not yet been applied as a natural antioxidant, mainly due to the complex heterogeneous structure and polydispersity of lignin. This report presents antioxidant capacity studies completed using the 2,2-diphenyl-1-picrylhydrazyl (DPPH) assay. The influence of purification on lignin structure and activity was investigated. The purification procedure showed that double-fold selective extraction is the most efficient (confirmed by ultraviolet-visible (UV/Vis), Fourier transform infrared (FTIR), heteronuclear single quantum coherence (HSQC) and ^31^P nuclear magnetic resonance spectroscopy, size exclusion chromatography, and X-ray diffraction), resulting in fractions of very narrow polydispersity (3.2–1.6), up to four distinct absorption bands in UV/Vis spectroscopy. Due to differential scanning calorimetry measurements, the glass transition temperature increased from 123 to 185 °C for the purest fraction. Antioxidant capacity is discussed regarding the biomass source, pulping process, and degree of purification. Lignin obtained from industrial black liquor are compared with beech wood samples: antioxidant activity (DPPH inhibition) of kraft lignin fractions were 62–68%, whereas beech and spruce/pine-mixed lignin showed values of 42% and 64%, respectively. Total phenol content (TPC) of the isolated kraft lignin fractions varied between 26 and 35%, whereas beech and spruce/pine lignin were 33% and 34%, respectively. Storage decreased the TPC values but increased the DPPH inhibition.

## 1. Introduction

### 1.1. Lignin Availavility and Structure

**Figure 1:**
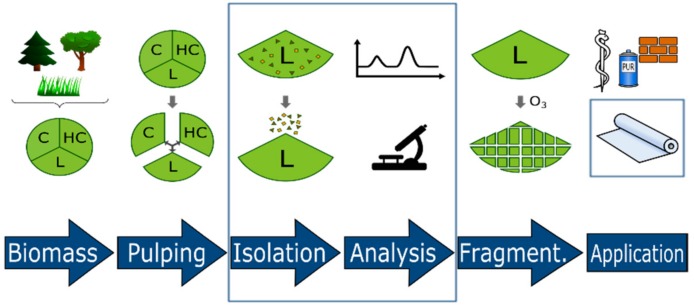
Development of lignin-derived materials starting with biomass pulping, lignin isolation and structure elucidation, guided fragmentation (e.g., ozonolysis), and application in construction, packaging, and biomedicine (C: cellulose, HC: hemicellulose, L: lignin).

The main components of lignocellulosic feedstock (LCF) are cellulose, hemicellulose, and lignin (Figure 1). Lignin, a multi-substituted phenolic polymer, forms 15–30 wt % of dry LCF, accounting for up to 30% of the organic carbon on earth. Roughly 10 years ago, the first biorefinery concepts were reported in Europe [1,2]. Today, lignin is intensively studied as one of the most promising biorefinery platform candidates that could be used for aromatic chemical production and energy supplementation [3,4,5,6].

The main function of lignin is to provide strength and mechanical support to the plant [7]. It is partially interconnected and forms a tight structure resistant to the influences of solvents and heat. Therefore, biorefinery concepts require efficient fractionation of the ligno-carbohydrate complex. Several methods to remove lignin from the biomass were developed including sulfate (kraft) and sulfite pulping [8], organosolv [9], alkaline polyol [10], and several steam explosion processes [11].

The biosynthetic precursors are composed of three phenylpropanoid units (coniferyl, sinapyl, and *p*-coumaryl alcohol) that, by various oxidative coupling reactions, form a randomly cross-linked macromolecule with different inter-unitary linkages. The structural building blocks are joined together by ether linkages and carbon-carbon bonds, and are consistent with the close association between lignin and hemicelluloses in the wood cell wall. There are also chemical bonds between these constituents [12].

**Figure 2:**
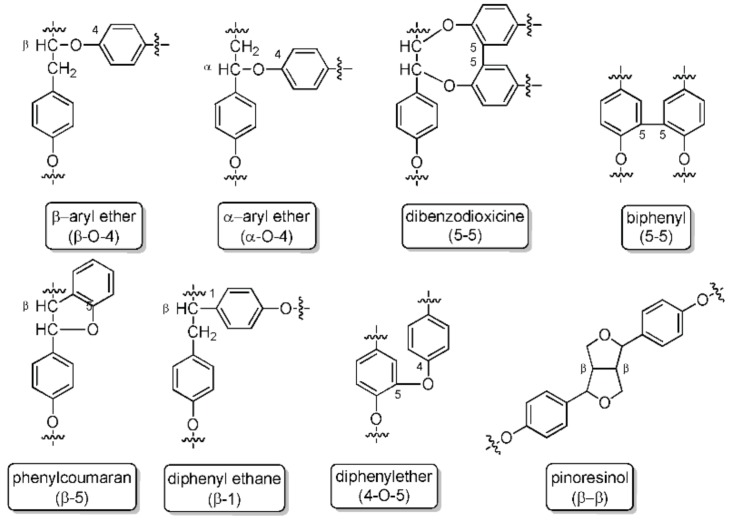
Lignin linkages: ether bonds, carbon-carbon bonds, and more complex linkages.

The removal of lignin by kraft delignification is achieved by treating wood material in an aqueous solution of sodium hydroxide and sodium sulfide. Kraft lignin (KL) has several characteristic properties that distinguishes it from native and other technical lignin: kraft lignin contains a greater number of phenolic groups due to the extensive cleavage of β-aryl bonds during kraft pulping, some biphenyl units, and other condensed structures, as a result of the severe cooking conditions (Figure 2) [13].

Due to the low selectivity of kraft pulping, black liquor from this process contains a significant amount of carbohydrate-derived substances, mainly aliphatic carboxylic acids [14,15]. Lignin, as a source of phenolic units, is one of the most lucrative candidates for various applications, e.g., as an emulsifier, adsorbent, carbon fiber precursor, antioxidant, and co-reagent in phenol-formaldehyde resins and thermoplastics [16,17]. However, lignin valorization is challenging due to its randomly-linked monolignol units, resulting in a complex and irregular chemical structure. Thus, improving lignin-derived materials for use in industrial applications has been limited to very few examples. The reproducible quality of the isolated structures requires considerable effort. Sequential depolymerization via oxidative or reductive methods is one of the favored approaches used to generate well-defined lignin fragments, which was comprehensively reviewed by Schutyser et al. [18]. However, any additional chemical treatment incurs increased costs.

### 1.2. Antioxidant Capacity and Corresponding Assays

As a polyphenol, lignin has the potential as an antioxidant to prevent oxidation reactions in biofuels, animal feeds, and polymeric composite materials. The complex structure of lignin, composed of aromatic rings with hydroxy and methoxy functional groups, is responsible for this antioxidant potential, primarily due to the termination of the oxidation propagation reaction through hydrogen donation and single electron transfer reactions. The application of technical lignins as natural antioxidants has not been implemented in the industrial sector, mainly due to the highly non-homogenous, complex structure and high polydispersity of lignin. Typically, purification and fractionation steps are necessary to enhance its stabilizing effect [19].

To determine the strength of an antioxidant, its ability to scavenge radicals or its reducing power are measured quantitatively. Several assays to determine the antioxidant capacity were reported for vanillin and corresponding derivatives in Garrett et al. [20]. When reviewing the published studies on the antioxidant activity of vanillin, Tai et al. concluded that they are not consistent between assays. Thus, they systematically evaluated the antioxidant activity of vanillin using multiple assay systems. Vanillin showed stronger activity than ascorbic acid and Trolox in the 2,2′-azino-bis(3-ethylbenzo thiazoline-6-sulphonic acid (ABTS) scavenging assay but showed no activity in the 2,2-diphenyl-1-picrylhydrazyl (DPPH) radical- and galvinoxyl radical-scavenging assays. Vanillin showed much stronger activity than ascorbic acid and Trolox in the oxygen radical absorbance capacity (ORAC) assay. In ABTS and ORAC assays, vanillin reacts with radicals via self-dimerization, contributing to the high reaction stoichiometry against ABTS·+ radical cations and 2,2′-azobis(2-methylpropionamidine) dihydrochloride (AAPH)-derived radicals [21,22]. The most important assays used for lignin studies are presented in Table 1.

Assays can be divided in two mechanisms: the hydrogen atom transfer (HAT) mechanism, where radicals are quenched by hydrogen atom donation of the antioxidant, and the single electron transfer (SET) mechanism, where the antioxidant´s ability to transfer one electron to reduce any compound is used, as shown in Equations (1) and (2), respectively.

HAT: X· + AH → XH + A(1)

SET: X· + AH → X^−^ + AH^+^ (2)

where X· is the radical, and AH is the antioxidant resulting in protonated radical XH and antioxidant radical, or anion X and radical cation AH·^+^.

Often, both mechanisms occur in parallel, resulting in complex reaction kinetics and numerous side reactions. The most relevant criteria for determining the mechanism and the efficacy of antioxidants are their bond dissociation energy and ionization potential. Polyphenols, as lignins, possess multiple activities. Thus, their antioxidant activity depends on the medium and corresponding solubility as well as testing substrate.

The ORAC assay is based on the interaction of the peroxyl radical ROO· (R = alkyl, aryl) with fluorescein. ORAC values are usually reported as Trolox equivalents with the help of a standard curve for measurements of Trolox samples of different concentrations. Ponomarenko et al. studied the fractionation of soft and hardwood LignoBoost kraft lignins, using sequential extraction with organic solvents, and reported that all fractions showed good results in the ORAC assay at the level of Trolox or even better [20,22]. In the Ferric Reducing Antioxidant Power (FRAP) assay, reduction of ferric 2,4,6-tripyridyl-*s*-triazine (TPTZ) to a colored product is measured photometrically [Fe(III)/Fe(II)] [23]. Antioxidant compounds with a redox potential below 0.7 V can be detected. The Cupric Reduction Antioxidant Capacity (CUPRAC) assay is a variant of the FRAP assay, using a CU(II)/CU(I) reduction which makes this assay more selective than the FRAP assay due to the lower redox potential [24]. So, sugars (potential residuals of biomass pulping) are not detected by this assay. The 2,2′-azino-bis(3-ethylbenzo thiazoline-6-sulphonic acid (ABTS) assay is also based on a redox reaction of the ABTS radical cation. Results are expressed relative to Trolox [25,26]. Analogously, the DPPH assay uses the redox reaction of 2,2-diphenyl-1-picrylhydrazyl (DPPH) with an antioxidant, resulting in reduced color intensity proportional to the antioxidant concentration [27]. The Folin Ciocalteu Assay or Total Phenolics Assay is used to measure the total phenol content (TPC) of natural products years. The Folin-Ciocalteu reagent (a mixture of phosphomolybdate and phosphotungstate) reacts with an antioxidant, changing the color intensity proportionally to the antioxidant concentration. Gallic acid is used as a reference compound and results are expressed as Gallic Acid Equivalents (GAE) or TPC [28].

Comparing these assays, it is important to identify the type of radicals that are crucial for in vivo conditions, i.e., using lignin as antioxidative additive in food, cosmetics, or biomedicine [29,30]. Here, the ORAC assay is best, since peroxyl radicals ROO· are more related to in vivo conditions than DPPH− and ABTS+ radicals due to their size. Thus, steric hindrance of DPPH− and ABTS+ radicals influence the reaction kinetics. In addition, results of the ORAC assay are independent of the reactivity rate in the antioxidant/substrate system. Conversely, the evaluation of FRAP, CUPRAC, ABTS, and DPPH assays require chosen reaction end points. However, these end-points usually do not represent the exact potential of the antioxidant.

The antioxidant activities of lignin samples have mainly been studied using the DPPH assay and the Folin-Ciocalteu method. Dizhbite et al. developed structure-property relationships regarding antioxidant activity, proposing that the π-conjugation systems of lignin operate as catalysts/activators in the interaction with DPPH radicals, and heterogeneity and polydispersity critically decrease the antioxidant efficiency. Using electron paramagnetic resonance (EPR) spectroscopy to characterize paramagnetic polyconjugated clusters in lignin samples, paramagnetic polyconjugated clusters were confirmed to result in a linear increase in antioxidant capacity, whereas aromatic OH and OCH_3_ contents were less influential [30]. Santos et al. studied isolation and purification effects including solvent influence, comparing water and organic solvents versus alkaline solution. They found that lignins with a low percentage of phenols showed the highest elimination of DPPH radicals [31].

## 2. Results and Discussion

### 2.1. Lignin Structure Analysis

**Figure 3:**
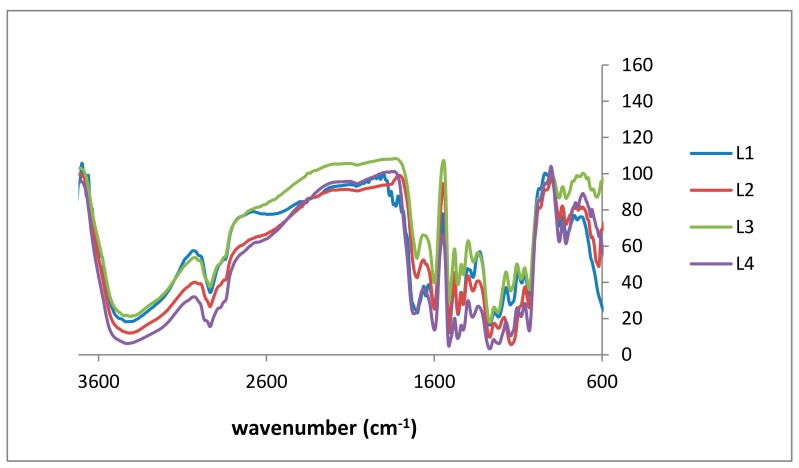
Fourier transform infrared (FTIR) spectra of lignin purification fractions: L1 (blue), L2 (red), L3 (green), and L4 (purple).

Structural analysis to specify accessible functional groups is essential to classify lignins for further study of antioxidant capacity and related structure-property relationships. Independent of the used renewable resource, Fourier transform infrared (FTIR) spectra of lignin show the vibrations characteristic for the guaiacyl (G) unit with intensity deviations due to the biomass, i.e., G ring and C=O stretching around 1260 cm^−1^, and C–H out-of-plane vibrations in positions 2, 5, and 6 around 850 cm^−1^ (Figure 3) [32]. In Table 2, the signals of the isolated kraft lignin fractions are listed and assigned confirming literature data [33,34]. L4 has the broadest OH stretching peak due to having the fewest impurities. The broadness of this peak decreases when in a reverse order (i.e., from L4 to L1). The spectra show fewer impurities as well as less noise: the signal ratio from L1 to L4 confirms the purification procedure.

Regarding antioxidant activity, aromatic hydroxy groups are of specific interest, although the significance of aliphatic groups has not yet been finally clarified. Usually, aliphatic and aromatic OH signals are observed at 3415 and 1265 cm^−1^, respectively [34,35]. In addition to literature data, a sharp signal of C–H out-of-plane in *m*-position of guaicyl units was observed. Figure 3 shows the FTIR spectra of all four lignin fractions.

Boeriu et al. evaluated FTIR spectroscopy to estimate the chemical composition and functional properties of various lignin samples using multivariate analysis. The authors correlated chemical composition and antioxidative properties with the FTIR spectral data [36]. Partial least squares (PLS) models were used to predict the major components’ concentrations and radical scavenging activity at the 99% confidence level, presenting *R*^2^ values higher than 0.80 in most cases. Analogous to these studies, we used FTIR data and principal component analysis (PCA) in order to specify slight quantitative differences for lignins from different sources (non-wood and hard and soft wood), isolated using different processing technologies (kraft versus organosolv). Our findings were recently presented as a conference contribution and are soon to be published [37].

**Figure 4:**
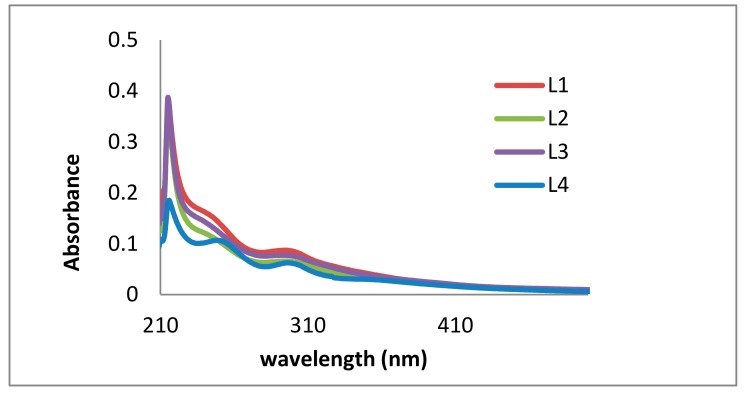
Ultraviolet-visible (UV/Vis) analysis of kraft lignin purification fractions dissolved in NaOH: L1 (red), L2 (green), L3 (purple), and L4 (blue).

Ultraviolet-visible (UV/Vis) studies (Figure 4) clearly show the difference between the lignin fractions. Significant improvement was achieved. L4 shows four distinct UV peaks (without any shoulders) due to π–π* and n–π* excitations of conjugated phenolic groups. In the literature, most of the spectra of kraft lignin contain at least two shoulders, as shown for the first three fractions (L1 to L3) of the purification procedure [37]. Due to the hypsochromic effect of NaOH, the main absorption (usually around 280 nm) shifts to 214–222 nm.

In Table 3, the two main UV/Vis absorption bands of lignin are shown and compared with literature data. The ester or ether bonds between acids, ferulic acids, and lignin were substantially cleaved by alkali treatment. The intensive absorbance at 279–280 nm relative to 316–320 nm indicates a high content of guaicyl (G) units, similar to that of other monocotyledons and is consistent with a guaicyl-rich lignin [38,39].

In 2015, Ponomarenko et al. published a comprehensive study summarizing spectroscopic data (i.e., UV/Vis) and antioxidant activity values of 50 different technical lignins of various botanical origins (e.g., annual plants, coniferous trees, and others), fractionated by different techniques (kraft process, fast pyrolysis, and hydrolysis). Antioxidant activity was studied using DPPH and ABTS assays [40]. The authors used chemometric methods, such as multivariate regression analyses, to explain structure-property-relationships. Thus, structure-related differences in antioxidant activity of lignins were quantified for the first time. They confirmed the positive influence of conjugated structural fragments within the lignin. This is in accordance with our findings showing four distinct absorption bands (instead of weak shoulders) for the purest fraction L4, having the stronger antioxidant activity (Section 2.2).

**Figure 5:**
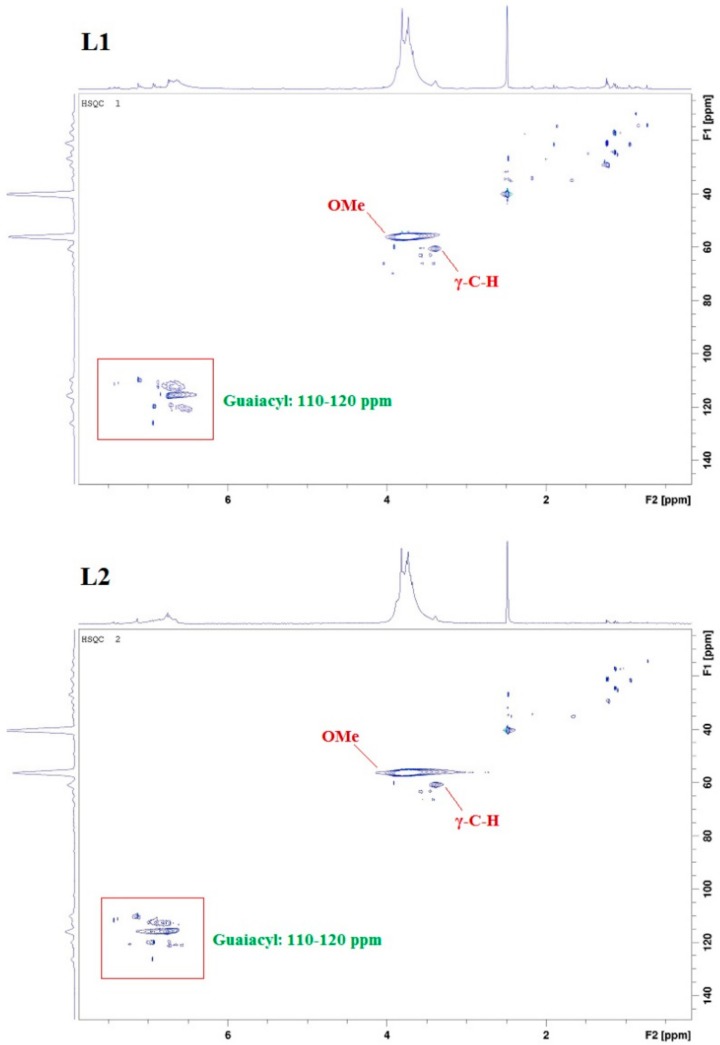

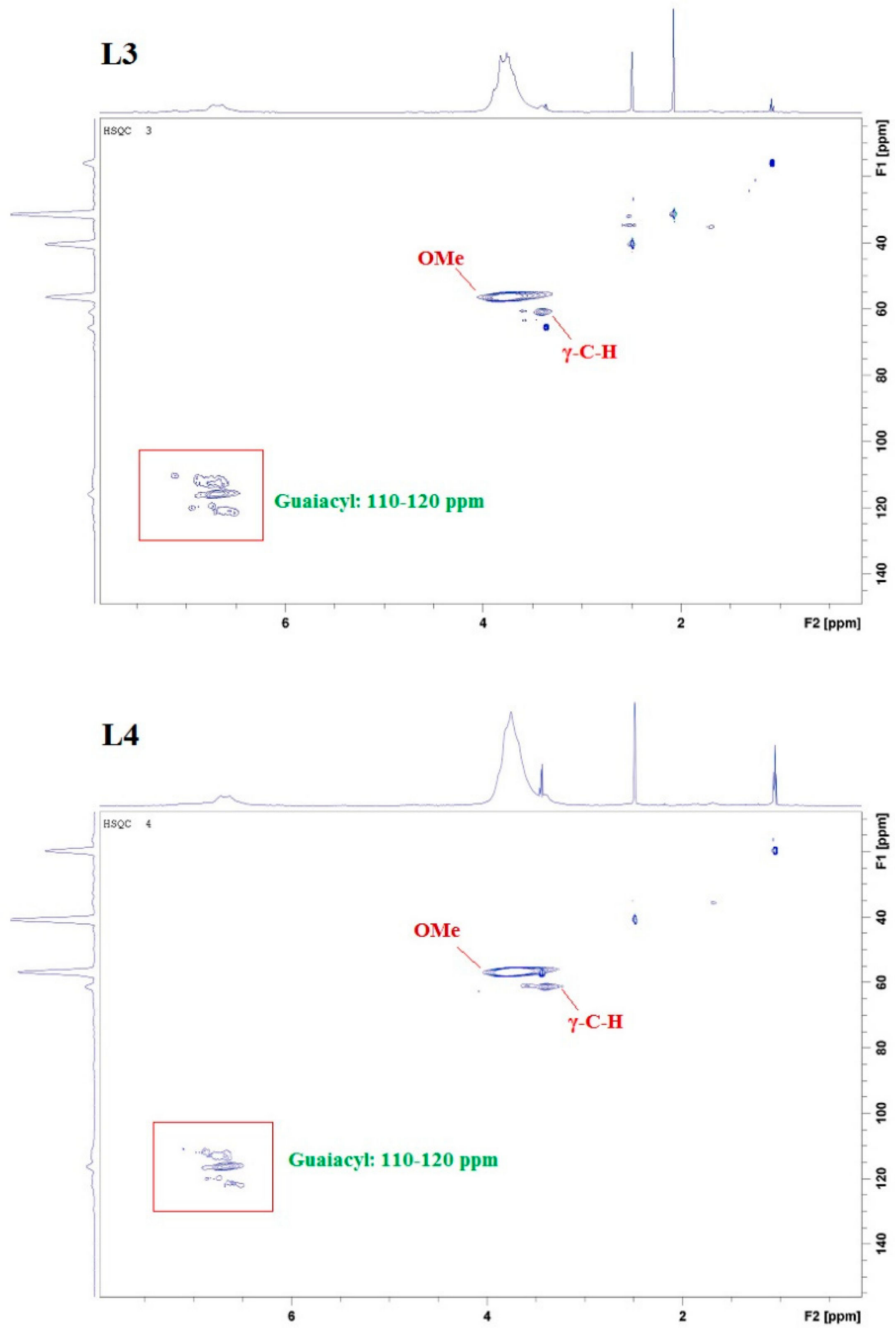
HSQC nuclear magnetic resonance (NMR) spectra of kraft lignin purification fractions L1, L2, L3, and L4; specifying in particular methoxy (OMe) signals, γ-C–H signals, and G-unit signals.

In addition to UV/Vis and FTIR spectroscopy, nuclear magnetic resonance (NMR) analysis (heteronuclear single quantum coherence (HSQC) and ^31^P) was used for structure elucidation. HSCQ NMR was applied to probe and specify the monolignol composition of lignins from different sources. The resulting ratios of syringyl/guiacyl/hydroxy-phenyl (S/G/H) were in close agreement with results obtained using pyrolysis gas chromatography-mass spectrometry (GC/MS) [14,18,19]. In our studies, three regions of lignin structure were identified via two-dimensional (2D) HSQC NMR (Figure 5): non-oxygenated and oxygenated aliphatic side chains appeared at δC/δH 50.0–90.0/2.5–6.0, and the aromatic region with C–H correlation signals at δC/δH 100.0–135.0/5.5–8.5 confirmed the literature data [13]. The following spectra exhibit intense signals at 56/3.7, corresponding to methoxyl groups and side chains in β-*O*-4- structures. A signal at 62/3.2 attributed to the gamma-C–H of gamma-acylated lignin units. A prominent region is located at 110–120/6.4–7, correlated with C–H aromatic signals from G units, whereas signals higher than 120 are related to aromatic C–H signals from H units.

**Figure 6:**
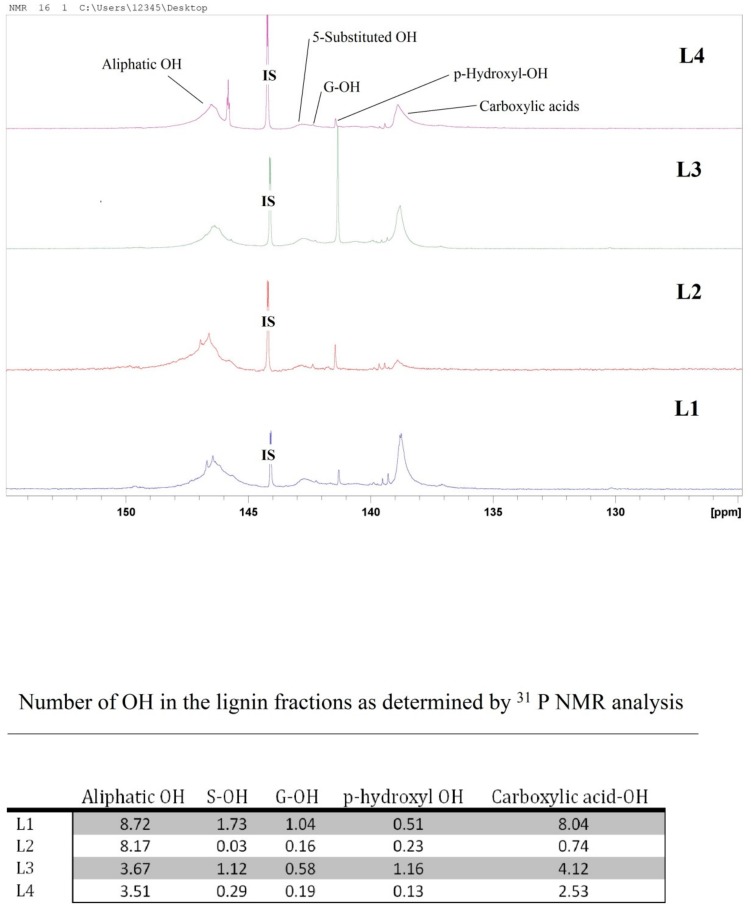
^31^P-NMR spectra of kraft lignin purification fractions: L1, L2, L3, and L4. The table shows the corresponding OH number in the fractions.

To specify phenolic OH groups, phosphorous derivatization is an appropriate and widely accepted method, described in detail by Argyropoulos and colleagues [41,42]. However, the sensitivity of a ^31^P-NMR experiment is about 15 times less than that of a proton (H^1^) NMR experiment, and the range of ^31^P chemical shifts is more than 1000 ppm for a variety of phosphorous compounds. The ^31^P-NMR spectra in Figure 6 show five OH regions for the lignin fractions: aliphatic OH between 151.0 and 144.7, 5-substituted OH between 144.0 and 142.3, guaiacyl OH between 142.5 and 141.5, *p*-hydroxyphenyl between 141.5 and 141.1, and carboxylic acids between 141.1 and 135.9. All lignin samples were phosphorylated with 2-chloro-4,4,5,5-tetramethyl-1,2,3-dioxa phospholane and analyzed via quantitative ^31^P-NMR spectroscopy with endo-*N*-hydroxy-5-norbornene-2,3-dicarboximide as the internal standard, according to the method described by Argyropoulos [42] and Sun et al. [43].

As shown in Figure 6, aliphatic OH’s dominate, except for L3 where carboxylic OH’s are more intense. In L1, the ratio of COOH–OH to aliphatic OH is almost 1:1, whereas aromatic OH forms almost half the aliphatic OH, indicating the presence of some small fragments or carbohydrates in L1. In L2, the aliphatic OH number is 11 times higher than the COOH. Most probably, carboxylic acid containing fragments are dissolved by the diethylether during extraction and discarded with the filtrate with some of the aliphatic OH-containing fragments. Extracting the lignin from L1 using acetone led to the loss of some aliphatic OH-containing compounds and the number of the aliphatic OH dropped to almost half. Those fragments are possibly small fragments that could be dissolved with acetone and discarded with the filtrate. In L4, where ethanol is used for the selective extraction of L3, the number of the COOH–OH’s dropped by half and the number of aromatic OH’s dropped by around five-fold. The number of aliphatic OH groups did not change significantly. Those results coupled with further analytical data, i.e., FTIR, UV/Vis, size exclusion chromatography (SEC), differential scanning calorimetry (DSC), and thermogravimetric analysis (TGA), proved the effect of the purification procedure.

In accordance with these results, Aminzadeh et al. reported ^31^P-NMR data for lignin fractions obtained from membrane filtration, showing that, despite the lower total content of phenolic OH groups, the low-weight average molecular weight (MW) sample had a higher proportion of non-condensed phenolic OH groups. The low-MW lignin fraction showed better antioxidant activity than the non-fractionated LignoBoost lignin in the kinetic ORAC test and demonstrated three-fold stronger inhibition of the substrate (fluorescein) than the reference antioxidant Trolox [44].

**Figure 7:**
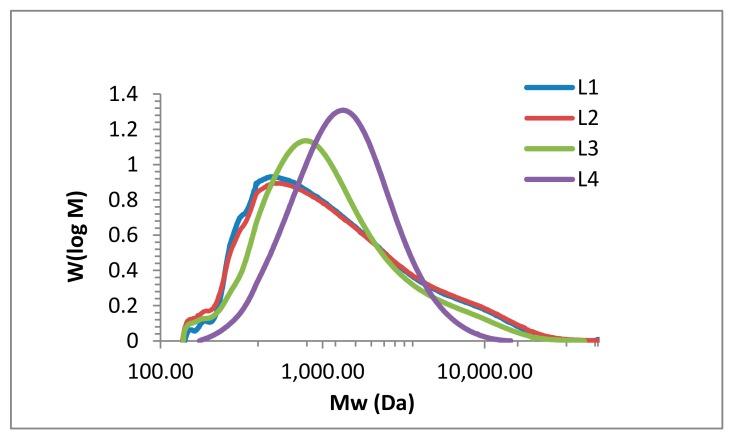
Size exclusion chromatography (SEC) analysis of kraft lignin purification fractions: L1 (blue), L2 (red), L3 (green), and L4 (purple).

The molar mass distribution is a key analytical parameter for technical lignin. Various approaches have been reported on how to address the molar mass distributions of lignin, mainly using SEC, viscosimetry, and light scattering analyses. The main drawback of SEC studies is the use of polymethylmethacrylate (PMMA) or polystyrene (PS) standards due to the lack of appropriate lignin standards. Both PMMA and PS do not represent the hydrodynamic volume of lignin. However, universal calibration approaches would require precise concentration data, which are also difficult to determine due to the poor solubility of lignin. A novel approach using SEC and HSQC NMR data combined with multivariate data analysis that enabled access to molecular weight and polydispersity data was reported for heparin [45]. In our studies using PMMA, the molecular weight of the isolated fractions varied from 877 to 6117 g mol^−1^, which corresponds to literature values for comparable technical lignins [12,30,46]. Figure 7 shows the SEC results of the four kraft lignin fractions (L1 to L4). In Table 4, the detailed values for the weight average molecular weight (MW), the number average molecular weight (Mn), and polydispersity (PD) for the lignin fractions are listed.

For all fractions, there is a clear dependency between purification and resulting SEC data. In L4, the spectrum shows one sharp peak with a maximum of 1157 g mol^−1^, whereas L1, L2, and L3 still show fragments and/or impurities of low and high molecular weight. The maxima of L1, L2, and L3 are 484, 466, and 878, respectively, with increasing intensity as the impurities decrease. Compared to literature data for kraft lignin (with values up to 6.500 g mol^−1^), all four fractions (L1 to L4) have a relatively low molecular weight. More importantly regarding the antioxidant activity, the polydispersity steadily decreases down to 1.6 for L4. In their review article, Espinoza-Acosta et al. discussed fraction procedures (organic solvent fractionation, differential precipitation, and ultrafiltration) and their influence on antioxidant activity, emphasizing the positive effect of decreasing polydispersity [47]. Argyropoulos reported the correlation of molecular weight antioxidant activity for various lignin fractions, confirming that lignin fractions of lower molecular weight possess higher antioxidant activity. Highest activity was observed for fractions of lowest polydispersity (PD of 3.7 in comparison to PD of 5.7 and 7, respectively) [48].

Lignin, being a three-dimensional (3D) amorphous polymer connected by phenylpropane structural units through β-*O*-4 ether and C−C linkages, contains a variety of reactive functional groups, in which methoxy is the most prominent group. According to the differences in the side chains, various structural fragments can be formed: guaiac wood-based lignin, lilac lignin, and hydroxyphenyl lignin, which can result in the formation of various phenolic compounds, such as 2-methoxyphenol, 4-methylguaiacol, and 2-methoxy-4-inylphenol, through propylene side chain splitting. The cleavage of the C−C bond in the guaiac wood-based lignin generates vanillin, whereas other products, such as ethers and alcohols, may be formed by unstable fracture of the long straight side chain in the complex macromolecular structure of lignin.

**Figure 8:**
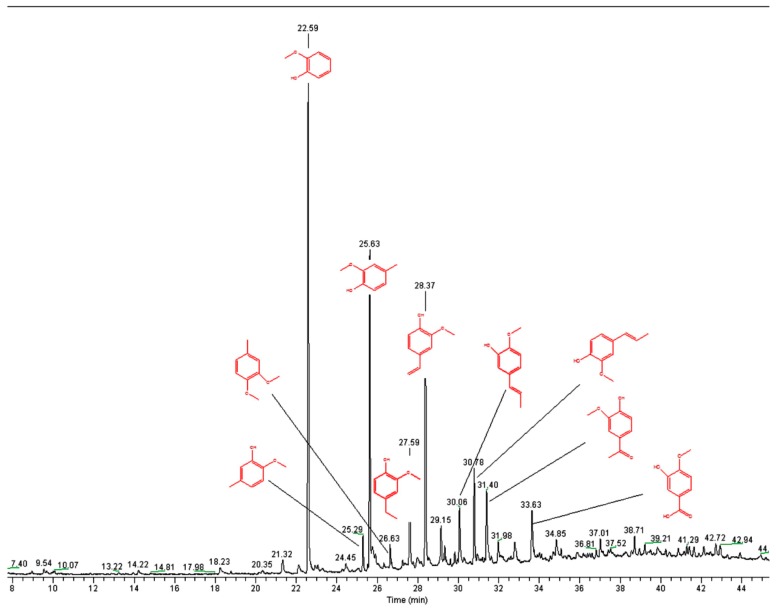

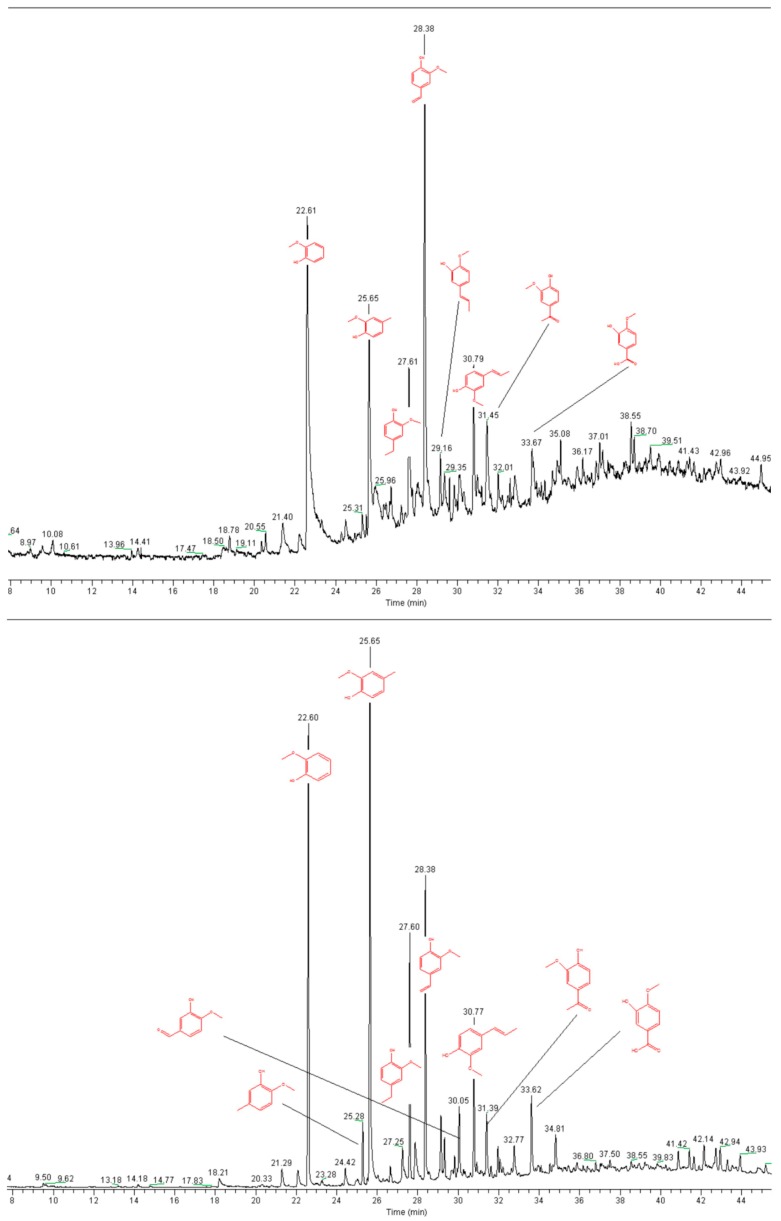

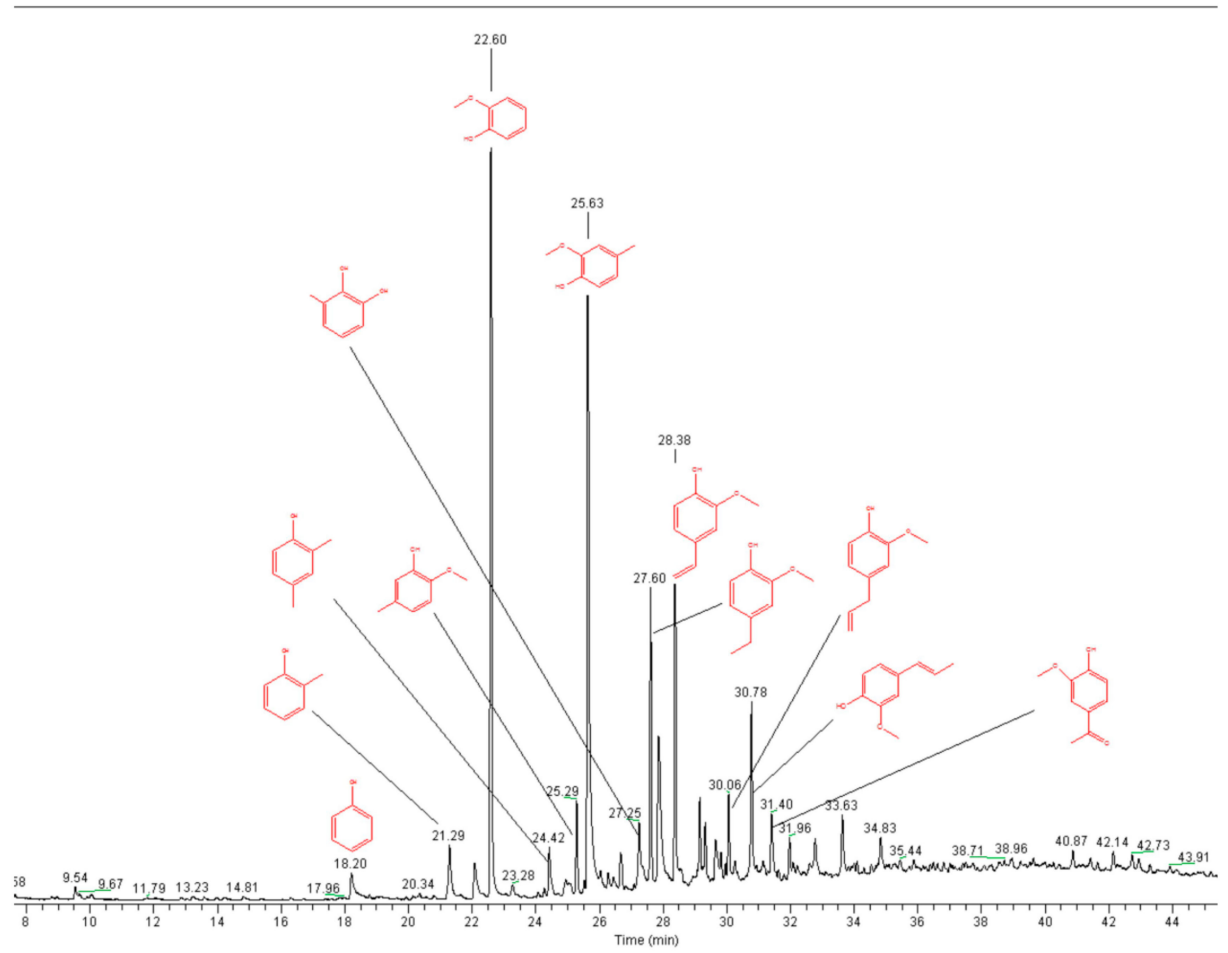
Pyrograms of kraft lignin purification fractions: (**a**) L1, (**b**) L2, (**c**) L3, and (**d**) L4 (measured at 550 °C).

Although lignin only has three basic structural units, the reactivity of the functional groups at the aromatic rings of each basic structural unit is different. This results in a highly complex pyrolysis process of lignin [49]. Figure 8a–d show the Py-GC/MS chromatograms for the fractions L1, L2, L3, and L4, respectively, with the assignment of the main signals.

**Figure 9:**
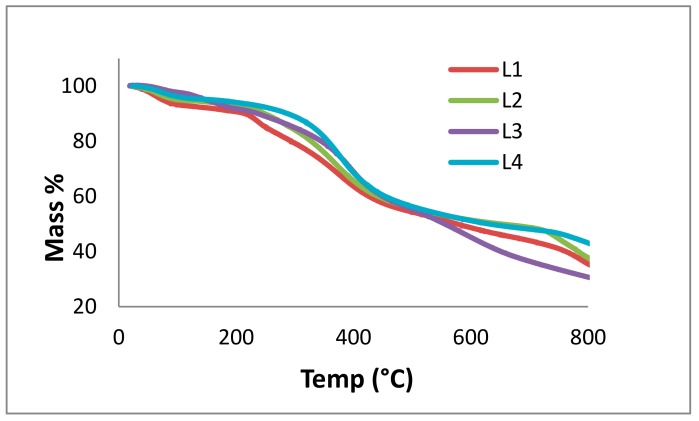
Thermogravimetric analysis (TGA) curves for kraft lignin purification fractions: L1 (red), L2 (green), L3 (purple), and L4 (blue), measured from 0 to 800 °C.

TGA is used to determine the mass loss of samples due to temperature treatment, indicative of thermal stability and thermal decomposition of a compound. Here, TGA of the lignin fractions was measured according to a procedure used by Vallejos et al. (Figure 9) [50]. The L1 TGA curve shows a complex decomposition process that resulted from five overlapping steps with the main maximum of mass loss rate at 60, 240, 380, 790, and 880 °C. The total mass loss was 82.08 wt %. L2 decomposed in four steps at 60, 372, 780, and 880 °C. The total mass loss was 76.90 wt %. The decomposition in L3 occurred in four steps: 60, 150, 395, and 900 °C. The total mass loss was 99.79 wt %. L4 decomposed at 60, 377, 810, and 900 °C. The total mass loss was 99.91%. In thin layer chromatography (TLC), spotting of the lignin fractions occurred at 25, 40, 60, and 90 °C; a new spot appearing for the fractions at 60 and 90 °C can likely be assigned to a fragment in the mobile phase, not necessarily evaporating water.

The first weight decrease (up to 259 °C) is ascribed to the moisture content in the lignin and the release of volatile products, such as carbon monoxide and carbon dioxide. Creation of vinyl guaiacol, ethyl, and methyl by-products is usually observed between 230 and 260 °C with the degradation of the propanoid side chains of lignin [31,32]. In the temperature range of 160 to 270 °C, thermal treatment of lignin was followed by condensation processes and led to the formation of unsaturated C=C bonds. The major decomposition of lignin structure occurred in the range of 260 to 478 °C. At temperatures lower than 310 °C, cleavage of aryl ether links occurred due to its low thermal stability. Eventually, the final stage occurred above 478 °C, which involved the formation of char residues [51].

**Figure 10:**
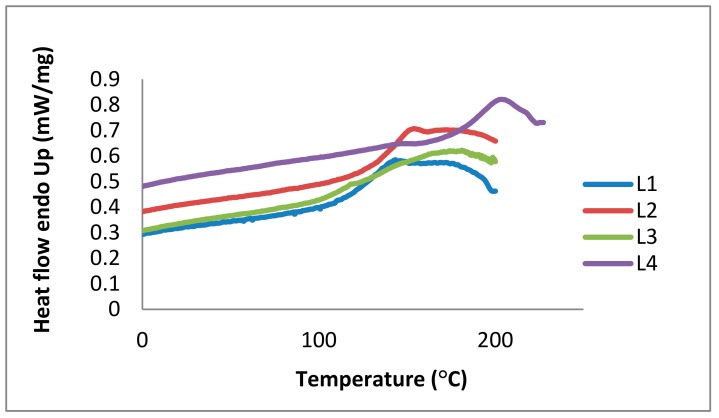
Differential scanning calorimetry (DSC) curves of the kraft lignin purification fractions L1 (blue), L2 (red), L3 (green), and L4 (purple).

In addition to TGA, DSC is the most common method used to determine thermal behavior and glass transition temperatures (*Tg*) of polymers and is also used for lignin analysis [13,32,34]. The glass transition (a reversible phenomenon) is correlated with the viscoelastic behavior of amorphous polymers. At temperatures below the glass transition, viscoelastic materials are stiff and glassy. This stiffness decreases in the transition region; the material shows a rubber-like elasticity resulting from chain entanglements. Glass transitions are correlated with local rotational or translational flowing of molecular segments at increasing temperatures. Thermal expansion increases the free volume of lignin. In particular, softwood kraft lignins show higher *Tg* values than organosolv lignins derived from hardwoods [32]. The *Tg* of lignin is affected by factors such as presence of low molecular weight contaminants (including water and solvents), MW, thermal history, and crosslinking. DSC measurements show that the *Tg* of L4 has the highest value at 185 °C, 140 °C for L2, 125 °C for L3, and 123 °C for L1. In accordance with literature data, these *Tg* values are associated to hydrogen bonds between hydroxyl groups and to the aromatic lignin nature (Figure 10).

Thermal analysis shows that lignin fractions start to decompose at temperatures around 60 °C, followed by other decomposition stages at higher temperatures. This means that the structures of the lignins change in physical state due to partial decomposition. In 2011, Reda et al. reported antioxidant stability results using thermal analysis, confirming a protective effect of lignin in heated edible vegetable oils [52]. In our study, DSC and TGA were required to describe the thermal stability of the different lignin fractions (L1 to L4). Partial decomposition due to thermal impact explains storage effects (Section 2.2.1). In terms of applications, the purified lignins could be used at temperatures below 60 °C to avoid thermal decomposition.

**Figure 11:**
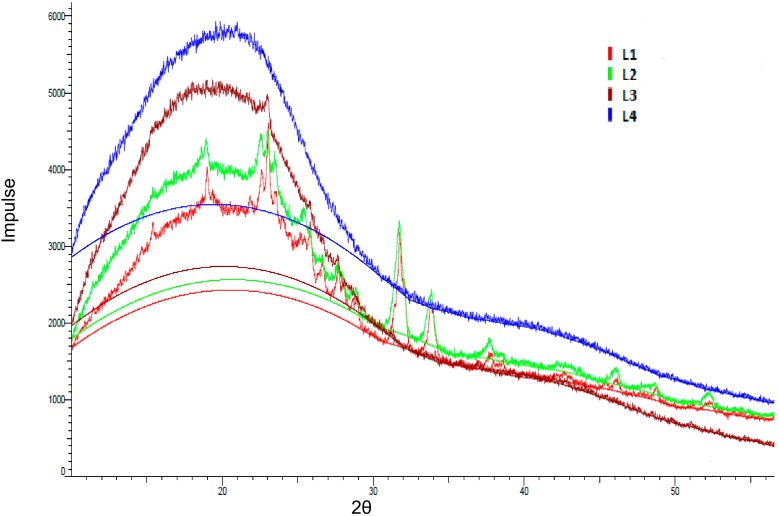
X-ray diffraction (XRD) diffractogram of the kraft lignin purification fractions L1 (red), L2 (green), L3 (brown), and L4 (blue).

X-ray diffraction (XRD) studies were performed to study the lignin morphology in more detail. All lignin fractions showed a broad diffraction of amorphous halo with a maximum at about 2θ = 20 °C (Figure 11). In fractions L1, L2, and L3, there were some sharp peaks indicating a certain crystallinity due to impurities and/or small crystalline fragments as discussed for the SEC results. L4 showed an amorphous pattern with no sharp peaks at all. The intensity increased with the purification level from L1 to L4. As the crystallite signal size decreased, the diffraction peaks broadened. Once the size was sufficiently reduced, the crystalline diffraction peaks broadened to the extent that they merged into each other, forming a single broad diffraction peak (the blue halo) supporting the amorphous nature of lignin and proving the effect of the purification procedure (Figure 11).

Analogue to our results, Dos Santos et al. precipitated lignins at different pH conditions with hydrochloric and sulphuric acid, respectively, and used X-ray diffraction in order to study the purity. The structure of lignins showed a variable composition according to the acid and the pH value. In detail, a crystalline phase was detected in precipitated kraft lignins assigned to sodium sulphate and sodium chloride salts formation [53]. In conclusion, the structural analysis of all four fractions could confirm the stepwise purity improvement obtained via sequential extraction.

### 2.2. Antioxidant Activity and Total Phenol Content

The antioxidant activity of the lignin fractions was evaluated using the DPPH method according to studies reported by Santos et al. [30]. The reactivity of DPPH is far lower than that of oxygen-containing free radicals (OH, RO, ROO, and O_2_), and unlike them, the interaction rate is not diffusion-controlled. Rather good conformity of the results obtained using the DPPH and ABTS methods respectively has been reported [4]. As their free radical scavenging ability is facilitated by their hydroxyl groups, the total phenolic content is used to rapidly screen antioxidant activity, measured as chemical reducing capacity relative to gallic acid. Here, the TPC was determined using the Folin-Ciocalteu reagent. Table 5 shows the DPPH inhibitions of lignin fractions compared to organosolv lignin obtained from beech (DL) and organosolv lignin obtained from spruce and pine (OLSW).

All kraft lignin fractions exhibited higher DPPH inhibition compared to literature data of kraft lignin isolated under same conditions. L4 (extracted from ethanol) produced the highest among them and L3 (extracted from acetone), the least. Variations in the antioxidant capacity of different fractions were mainly attributed to differences in their phenolic content and the type of phenolics, which, in turn, depends on the solvent used for the extraction [54].

In this study, the DPPH inhibition was affected by the polarity of the extraction solvent. Thus, the trend in DPPH inhibition of lignin fractions is ethanol > diethylether > acetone. Here, there were two factors affecting the activity: the presence of fragments and the solvent polarity. In the first two fractions (L1 and L2), the number of impurities was higher than in L3 and L4. Those impurities could include small molecular weight phenols (monomers) THAT exhibit a certain antioxidant activity. Secondly, the extraction solvent polarity significantly influences the amount of accessible phenolics responsible for the antioxidant activity.

The TPC values of the kraft lignins studied are between 26.8 and 35%, whereas Santos et al. reported maxima of 29.61% [31]. The TPC values show that purifying the lignin starting from L1 to L4 increased the phenolic content (except for L2 where diethyl ether was used to soak). For L4, the last extraction fraction with the highest purity showed the highest TPC value. The solvent used in the purification significantly affected the TPC data, as reported in studies of polyphenol extraction from different plants, confirming a proportional effect of solvent polarity and TPC value [55,56]. Here, we confirm those results for solvent extraction using ethanol, acetone, and diethyl ether. Diethyl ether, being the least polar among them, had a negative effect on the TPC of the extracted lignin, and ethanol, being the most polar, had a positive effect. Organosolv-derived lignins (OLSW and DL) possess a higher TPC compared to kraft lignins, indicating that the organosolv process maintains a reasonable amount of the phenolic structure in the lignin. As reported in various studies, there is no clear correlation between DPPH inhibition and TPC values, most probably due to the type of phenolics responsible for the antioxidant activity [57].

Using another assay (ABTS), García et al. studied the effects of processing parameters on the lignin antioxidant activity. It was shown that lignin, though at higher amounts, could attain the same level of antiradical activity as some powerful and well-known commercial antioxidants, such as Trolox [58,59]. Analogous to our studies, Kaur and Uppal investigated the capacity of lignin derived from sugarcane bagasse in the reduction of DPPH radicals. They found that the antioxidant activity of lignin was higher than that of 3,5-di-tert-butyl-4-hydroxytoluene (BHT) but was lower than that of 3-tert-butyl-4-hydroxyanisole (BHA), and concluded that sugarcane bagasse lignin has the potential to be used as an antioxidant in food oils and fats [60]. According to Sun et al., antioxidant strength of lignin is mainly caused by methoxyl groups in the ortho position, stabilizing phenoxyl radicals by resonance as well as hindering radical propagation [61]. Additional stabilization is provided by conjugated double bonds through extended delocalization, which is in agreement with highest values for the L4 fraction, showing four distinct absorption signals in UV/Vis spectroscopy (Section 2.1). In addition to BHA and BHT, Gadioli et al. compared lignin with industrial Irganox 1010 as the primary stabilizer in formulations for polypropylene. The authors confirmed the better antioxidant performance of lignin due to its cross-linking macromolecule nature [62]. So, regarding the application perspective, lignin is a promising candidate to be evaluated in more detail as an antioxidant additive in polymers, food, and cosmetics.

#### 2.2.1. Source, Storage, and Temperature Effects

**Figure 12:**
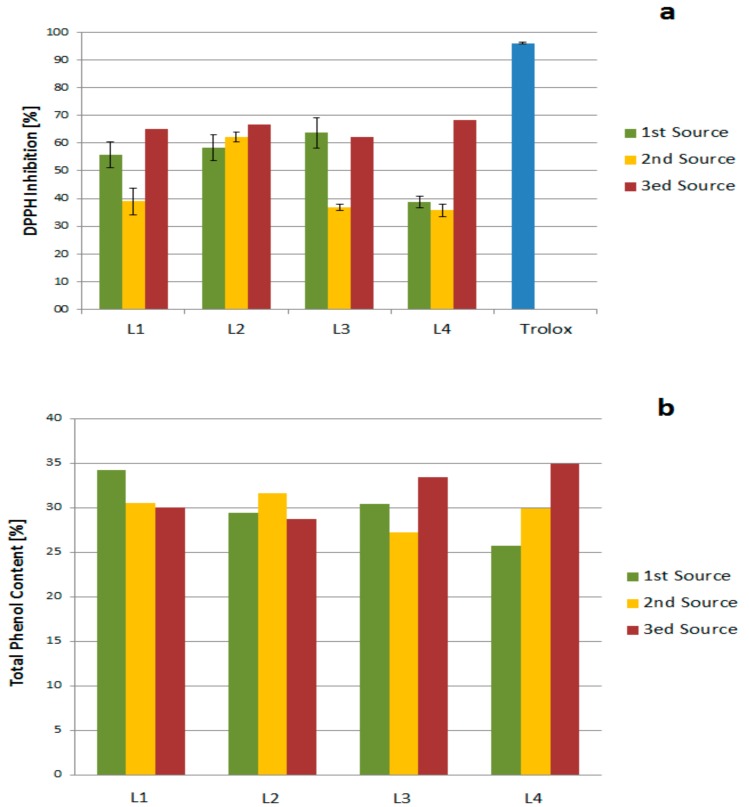
Source effect on (**a**) DPPH inhibition and (**b**) Total Phenol Content (TPC).

Three different batches of black liquor were used for the extractions. The products of those extractions were grouped into three sets, each representing one source. The analytical characterization of those three sets of fractions showed slight differences in the lignin structures. DPPH inhibition and TPC of the three sets were completed (Figure 12) and showed deviations without clear correlations or trends. This could be caused by differences in the harvesting time of the biomass, the age of the biomass used for the pulping, and/or the percentage of the spruce/pine in the biomass pulped.

**Figure 13:**
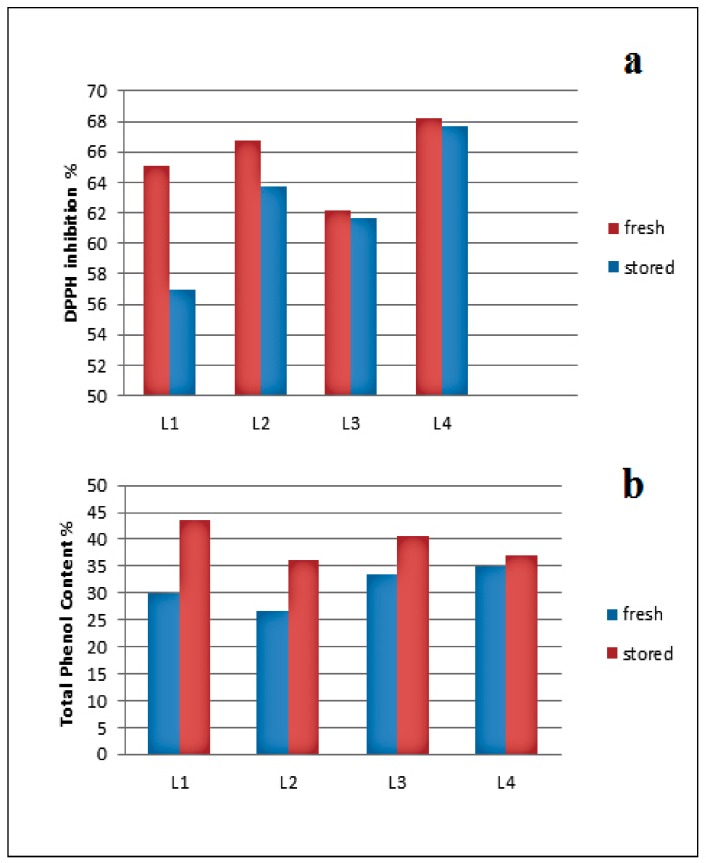
(**a**) Storage effect on DPPH inhibition of kraft lignin purification fractions (fresh samples in red and stored in blue); (**b**) TPC where fresh samples are in blue and stored in red.

The effect of storage on the antioxidant activity and the TPC of the purification fractions was also investigated. The fractions were studied after purification, then stored for six months. DPPH inhibition and TPC were performed again for the stored fractions. The results (Figure 13) showed that the effect of storage on the DPPH inhibitions of the fractions decreased for all fractions. In contrast, the TPC of the fractions increased, likely due to hydroxyl formation during storage. The antioxidant activity was not affected. In ongoing studies, the structure of those degradation products will be analyzed.

**Figure 14:**
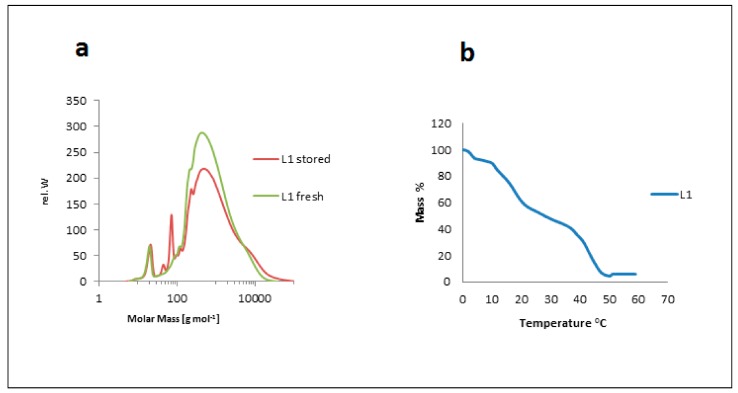
(**a**) Storage effect on lignin structure via SEC analysis of freshly isolated (green) versus stored (red) lignin (sample L1). (**b**) Temperature effect on the structure of lignin via TGA of kraft lignin (sample L1).

Degradation processes of the macromolecular lignin structure were not only affected by storage, but also by temperature and UV/Vis irradiation. Storing lignin purification fractions for 45 days at room temperature caused structural changes that were monitored by TLC. The solvent system used for the study was 90% ethanol and 10% *n*-hexane. L1 showed three spots: one dense spot at the baseline, which was the lignin spot; a second spot (small and light), located in the middle of the TLC sheet; and a third spot (large) traveling with the mobile phase. After 45 days of storage, TLC was repeated for the fractions. The spot in the middle for L1 looked denser and bigger than for fresh L1. This means that there were new fragment(s) in the lignin caused by degradation due to storage. SEC (or also gel permeation chromatorgraphy, GPC) analysis of both stored and fresh extracts showed this fragmentation clearly (Figure 14a). New peaks at smaller molecular weights (45 g mol^−1^ and 73 g mol^−1^) appeared for the stored samples. The fragmentation was neither caused by thermolysis since storage was performed at 25 °C nor by any chemical interaction. Samples were stored in aluminum foil-coated vials to prevent light from contributing to photolytic degradation, which otherwise would occur as observed and studied in detail for various lignins [37,63].

The samples were dried at different temperatures: 25, 40, 60, 70, and 90 °C. TLC spotting showed no change in L4 (one spot at 25 °C and at 40 °C), but a new spot for L4 traveling with the solvent system appeared at 60, 70, and 90 °C in addition to the lignin spot. The second spot in the middle of the TLC plate of the L1 fraction became denser and larger than in the dried fraction at 25 and 40 °C. TGA was measured for the lignin fraction according to Vallejos et al. [48]. The curve of L1 showed the temperatures for lignin degradation were mainly 60, 380, and 880 °C. The degradation temperatures for L4 were 60, 390, and 900 °C. Figure 14b shows the TGA curve of L1. Kraft lignin fractions were extracted from black liquor (a product of kraft pulping of softwood, mainly spruce and pine).

## 3. Materials and Methods

### 3.1. Isolation and Purification

Industrial black liquor was obtained from Zellstoffwerk Blankenstein GmbH (Blankenstein, Germany). Kraft lignin was extracted via gradual acidic precipitation of black liquor using HCl and H_2_SO_4_ at specific pH, temperature, and time of stirring variations. Results of these precipitation conditions were investigated for yield. The optimum acidification was the one using H_2_SO_4_ with stirring at room temperature and pH = 2 for 90–180 min to obtain the first fraction of lignin (L1). L1 was soaked with diethyl ether to produce the second fraction (L2). Selective extraction using acetone produced L3, another selective extraction of L3 using ethanol produced L4. Diethyl ether was used as a precipitating solvent for the selective extractions.

The purification was monitored by thin layer chromatography (TLC): the fractions were dissolved in dimethyl sulfoxide (DMSO) for the spotting and a mixture of 10% *n*-hexane and 90% ethanol was used as the mobile face.

Two organosolv lignins were prepared to be used for comparison studies: one from beech wood (DL) and one spruce/pine (OLSW) according to an earlier published procedure [32].

### 3.2. FTIR Analysis

FTIR spectra of the lignin samples were recorded on a Jasco FTIR 410 (Tokyo, Japan) spectrometer in the range of 3800 to 500 cm^−1^ using a KBr disc containing 1% finely ground samples. The spectrum recorded over 30 scans with a resolution of 4 cm^−1^.

### 3.3. UV/Vis Analysis

UV/Vis spectra were recorded on a Hewlett-Packard (Waltham, MA, USA) 450 Diode Array spectrometer. The lignin UV/Vis absorption spectrum was obtained at room temperature using a sample (3.2 mL) containing 50 µg mL^−1^ of kraft lignin (KL) in 0.1 M NaOH. The absorbance was measured in the range of 210 to 500 nm.

### 3.4. 2D HSQC NMR Analysis

HSQC spectra were measured by an NMR spectrometer Avance III 600 (Bruker, Karlsruhe, Germany) with 4 scans and 16 prior dummy scans. The data of 4000 points were recorded with a spectral width of 7211 Hz, a receiver gain of 2050, and a total acquisition time of 0.28 s. O1 was set to 5 ppm (^1^H) and 80 ppm (^13^C).

### 3.5. ^31^P NMR Analysis

^31^P NMR spectra were acquired using ^1^H-^31^P decoupling experiment (Avance III 600, Bruker, Karlsruhe, Germany) with 32 scans and 2 prior dummy scans. The data of 131,000 points were recorded with a spectral width of 12,175.324 Hz, a receiver gain of 2050, and a total acquisition time of 5.38 s.

### 3.6. SEC Analysis

The weight-average (Mw) and number-average (Mn) molecular weights of the lignins, as well as their polydispersity (PD) were determined by size exclusion chromatography (PSS SECurity2 GPC System, Mainz, Germany). Tetrahydrofuran (THF) was used as the mobile phase with a run time of 30 min and an injection volume of 100 µL. Polystyrene standards were used for the calibration at different molecular weights. The lignin sample was completely dissolved in THF (1 mg·mL^−1^) with gentle stirring at room temperature. Size exclusion chromatography was performed at room temperature with THF as the mobile phase (flow rate 1.0 mL·min^−1^) and UV detector (280 nm).

### 3.7. Pyrolysis GC-MS

Approximately 1 mg of lignin sample was inserted without further preparation into the bore of the pyrolysis solids injector and then placed with the plunger on the quartz wool of the quartz tube from the furnace pyrolyzer Pyrojector IITM (SGE Analytical Science, Melbourne, Australia). The pyrolyzer was operated at a constant temperature of 550 °C. The pressure of the helium carrier gas at the inlet to the furnace was 95 kPa. The pyrolyzer was connected to a Trace 2000 gas chromatograph (ThermoQuest/CE Instruments, Milan, Italy) with a quadrupole mass spectrometer Voyager (ThermoQuest/Finnigan, MassLab Group, Manchester, UK) operated in electron impact ionization (EI) mode. A fused silica GC capillary column DB-5 ms 30 m long, 0.25 mm I.D., 0.25 mm film thickness (J&W, Folsom, CA, USA) was used.

The gas chromatographic conditions were as follows: programmed temperature of the capillary column was from 60 °C (1 min hold) at 2.5 °C min^−1^ to 100 °C and then 10 °C min^−1^ to 280 °C (20 min hold at 280 °C). The temperature of the split/splitless injector was 250 °C and the split flow was 10 cm^3^·min^−1^. Helium, grade 5.0 (Westfalen AG, Muenster, Germany) was used as a carrier gas at a constant pressure of 70 kPa during the entire analysis. The transfer line temperature was 280 °C. The MS electron ionization (EI) ion source temperature was maintained at 250 °C. The ionization occurred with a kinetic energy of the impacting electrons of 70 eV. The current emission of the rhenium filament was 150 lA. The MS detector voltage was 350 V. Mass spectra and reconstructed chromatograms—total ion current (TIC)—were obtained by automatic scanning in the mass range of *m/z* 35 to 455 u. Pyrolysis-GC/MS data were processed with Xcalibur software (version 1.2, ThermoQuest, Milan, Italy) and the NIST 05 mass spectral library (Gaithersburg, MD, USA).

### 3.8. TGA

TGA was performed with about 10 mg of lignin using a Netzsch (Selb, Germany) TGA 209 F1 with a heating rate of 20 °C min^−1^ under nitrogen atmosphere. The temperature ranged from ambient to 800 °C.

### 3.9. DSC

Glass transition temperatures (*Tg*) were determined using a Netzsch Polyma (Selb, Germany) 8000 differential scanning calorimeter. The scans were run from a starting temperature of 0 °C (held for 3 min) under a nitrogen flow rate of 10 mL min^−1^. The samples were then heated from 0 to 226 °C at 20 °C min^−1^. Before being tested, the samples were extensively dried for 24 h in an oven at 50 °C under vacuum.

### 3.10. X-Ray Diffraction

Powdered lignin samples were used for obtaining XRD patterns. X-ray diffractograms with 2θ, ranging from 10° to 65° were collected with a Bruker D2 PHASER X-ray diffractometer (Karlsruhe, Germany) using theta/theta geometry with a secondary monochromator (CuKα radiation, 30 kV/10 mA, step 5407 in 2θ, 96 s/step).

### 3.11. Total Phenol Content

A volume of 2.5 mL Folin-Ciocalteu reactive and 5 mL of 20% Na_2_CO_3_ solution were mixed with 0.5 mL of lignin solution (20 mg in 10 mL of DMSO). The mixture was kept for 30 min at 40 °C before measuring the absorbance at 750 nm. The intensity of blue color was measured at 750 nm in a UV-VIS spectrophotometer (Jasco V-630, Silver Spring, MD, USA). The total phenols content was determined using a standard curve with gallic acid solutions.

### 3.12. Antioxidant Activity

Spectrophotometric method based on the use of the free radical DPPH using a Jasco V-630 spectrophotometer. Extracted samples dissolved in dioxane/water (90:10, *v/v*) at a concentration of 1 g/L; 0.1 mL of the sample solution was mixed with 3.9 mL of a 6 × 10^−5^ M DPPH solution, and the absorbance at 518 nm of the mixture was measured at 15 min and 30 min, respectively.

## 4. Conclusions

The antioxidant activity and related radical scavenging activity of lignin are correlated with a number of competing criteria: biomass phenotype and genotype, pulping and purification methods, and resulting heterogeneity. Here, the status quo of antioxidant assays, their advantages and limitations were shortly summarized. Antioxidant capacity studies using the DPPH assay show correlations between minor structural differences of the purified lignins. In detail, biomass source (beech versus spruce/pine), pulping process (kraft versus organosolv), and purification degree of the isolated lignins influence the antioxidant activity. The highest activity was found for the L4 fraction, which was the purest lignin with the most narrow MW distribution of 1.6 according to SEC analysis. A double-fold selective extraction was the most efficient purification procedure confirmed via spectroscopic and chromatographic methods (UV/Vis, FTIR, HSQC NMR, SEC, and pyrolysis GC-MS) and XRD analysis. Antioxidant activity measured via DPPH inhibition of the unmodified kraft lignin fractions was above the values reported in the literature, including commercial BHT, confirming that technical black liquor can be used without further modification.

Storage of the purified fractions decreased the TPC values and increased the DPPH inhibition. Further studies are required including multivariate data analysis in order to quantify storage effects in more detail and finally specify the structure-property-relationships most relevant for altering lignin antioxidant activity during storage.

## Figures and Tables

**Table 1:** Antioxidant assays, the corresponding reaction mechanism, advantages and disadvantages.

Antioxidant Assay	Mechanism	Advantages	Disadvantages	References
**ORAC** (Oxygen Radical Absorbance Capacity)	Hydrogen Atom Transfer	• can be adapted to detect both hydrophilic and hydrophobic antioxidants by altering the radical source and solvent • ORAC values account for lag-time, initial rate and total extent of inhibition in a single value • automation is possible	• fluorescence quenching is very sensitive, so any impurity has to be avoided • to achieve reproducible results, constant reaction conditions are required (temperature, pH, oxygen and reagent concentrations etc.) • detection requires fluorometer (fluorescence easy to be quenched) • analysis time about one hour • measurement is limited to peroxyl-radicals as oxidants	[20]
**FRAP** (Ferric Reducing Antioxidant Power)	Single Electron Transfer	• simple, quick, inexpensive, robust, does not require special equipment • direct method to measure the combined activity of multiple, reductive antioxidants in a sample • automation is possible	• no exact reaction time/reactivity is varying for different samples • thiol-containing antioxidants like glutathione are not detected	[23]
**CUPRAC** (Cupric Reduction Antioxidant Capacity)	Single Electron Transfer	• simple, quick, inexpensive, robust, does not require special equipment • all classes of antioxidants are detected, including thiols • applicable to both, hydrophilic and lipophilic antioxidants	• no exact reaction time/reactivity is strongly varying for different samples	[24]
**ABTS** (2,2’-azino-bis(3-ethylbenzothiazoline-6-sulphonic acid)	mixture of HAT/SET	• simple, quick, wide pH-range, often used • soluble in aqueous and organic solvents and not affected by ionic strength -> applicable to a wide range of hydrophilic and lipophilic antioxidants • several wavelenghts are available for photometric detection of the ABTS-radical	• no exact reaction time/reactivity is strongly varying for different samples • the bulky ABTS-radical is not a good model for small, biologically more relevant radicals like HO • etc.	[25]
**DPPH** (2,2-diphenyl-1-picrylhydrazyl)	mixture of HAT/SET	• simple, quick, often used, no special equipment needed • DPPH-radical is commercially available; no in situ-generation necessary	• no exact reaction time/reactivity is varying for different samples • DPPH-radical may have a poor reactivity with antioxidants due to its stability an sterical hindrance	[26]
**FC/TPC** (Folin-Ciocalteu-Assay or Total-Phenolics-Assay)	mixture of HAT/SET	• simple, often used, does not require special equipment	• no exact reaction time/reactivity is varying for different samples • interferences with other reductive substances may influence the results	[27]

**Table 2:** Fourier transform infrared (FTIR) functional group assignment of lignin fractions. The most important signals are listed with wavenumbers and signal assignment for all four fractions, L1 to L4 and compared with literature data of kraft lignin (KL).

L1 (cm^−1^)	L2 (cm^−1^)	L3 (cm^−1^)	L4 (cm^−1^)	KL Lit. [34,35] (cm^−1^)	Signal Assignment
3396	3408	3414	3396	3415	O–H stretching
2931	2931	2926	2925	2935	C–H stretching
2834	2814	2834	2833	2843	tertiary C–H group
1695	1695	1700	1702	1660	carbonyl-carboxyl stretching
1577	1583	1593	1595	1505	aromatic/carbonyl stretching
1452	1449	1455	1459	1451	C–H deformation
1263	1262	1265	1262	1265	C–O stretching, aromatic (phenyl)
1028	1028	1026	1028	1029	C–O deformation (methoxy group)
810	810	807	807	814	C–H out-of-plane in *m*-position of guaicyl units
848	848	848	848	-	C–H out-of-plane in *m*-position of guaicyl units

**Table 3:** Ultraviolet-visible (UV/Vis) absorption data of lignin and their characteristics.

λ exp. (nm)	λ Lit. (nm)	Functional Group	Intensity	Excitation	Reference
215–222	279–280	Non-conjugated phenolic groups (G/S rich)	high	π–π*	Azadi et al. [39]
296–303	316–320	Conjugated phenolic groups (*p*-coumaric acid, ferulic acid)	low	n–π*	Vivekanand et al. [38]

**Table 4:** Number average molecular weight (Mn), weight average molecular weight (MW), and polydispersity (PD) of lignin fractions L1 to L4 obtained from the size exclusion chromatography (SEC) analysis.

Fraction	Mn (g mol ^−1^)	Mw (g·mol^−1^)	PD
L1	720	2108	2.9
L2	706	2226	3.2
L3	757	1816	2.4
L4	1043	1690	1.6

**Table 5:** The DPPH inhibitions and total phenolic content (TPC) values of kraft lignin fractions (L1 to L4) and two organosolv lignins obtained from beech (DL) and spruce/pine (OLSW).

	L1	L2	L3	L4	DL	OLSW	Lit. [30]
DPPH inhibition (%)	65.1 ± 3.7	66.8 ± 6.6	62.2 ± 9.5	68.2 ± 3.6	64 ± 2.6	42 ± 1.9	54.76
TPC (%)	30 ± 1.2	26.8 ± 0.5	33.5 ± 0.9	35 ± 1.0	33.3 ± 1.6	34.1 ± 1.0	29.61

Values are mean ± SD of triplicate experiments. Trolox DPPH inhibition = 98% [30].
